# Molecular Identification of Infectious Enteropathogens in Faeces of Healthy Horses

**DOI:** 10.1177/11786361221089005

**Published:** 2022-04-11

**Authors:** Lisa Paruch, Adam M Paruch

**Affiliations:** Division of Environment and Natural Resources, Norwegian Institute of Bioeconomy Research (NIBIO), Aas, Norway

**Keywords:** DNA-based markers, equines, enteropathogens, qPCR, zoogenic faecal contamination

## Abstract

Zoogenic faecal contamination of the environment is one of the indices included in the evaluation of ecological threats, health hazards and adverse impacts on various ecosystems. The risks and environmental concerns are associated with the fact that faeces of wild and domesticated animals constitute the largest source of environmental loading of enteropathogens associated with transmission of zoonotic diseases (enteric zoonoses). Although sick animals are more likely to transmit pathogens, healthy ones can also be the carriers and defecate them into the environment. This is of particular importance given the close human-animal interactions and health effects resulting from human and ecological exposures to faecal hazards from companion and farm animals. We have therefore set out to investigate whether healthy equines can carry and defecate human infectious pathogens. For this purpose, we set up a pilot study to examine the faecal DNA of horses using culture-independent molecular diagnostics – fluorescent probe-based quantitative real-time PCR. Our results revealed that among a total of 23 horses, 6 were found to carry *Campylobacter jejuni* (*C. jejuni*), and 5 had *Salmonella enterica* serovar Typhimurium (*S.* Typhimurium). Moreover, *Enterococcus faecalis* (*E. faecalis*) was found in 14 horses, while 19 were positive for *Clostridium perfringens* (*C. perfringens*). Furthermore, the frequently reported protozoan parasites in livestock, *Cryptosporidium parvum* (*C. parvum*) and *Giardia lamblia* (*G. lamblia*), were discovered in 8 and 7 samples, respectively. This pilot study shed new light on the phenomenon of healthy horses carrying *C. jejuni* and other human-health-related enteropathogens.

## Introduction

Horses play vital role in humans’ lives, for example, in industry, agriculture, transport and, more recently, for leisure or sporting activity. There are nearly 60 million horses registered in the world.^1^ The last published data from Norway revealed up to 125 000 horses used mostly for trotting, racing, riding schools and boarding activities.^2^ They are historically and culturally embedded with Norway, and some municipalities even have horse symbols on their own coats of arms. Thus, horses have become fundamental components of the local landscape, life and society and interact with people frequently and intensively. It is very common to meet people, most often teens, on horses strolling/riding in the cities throughout the year.

Horses normally defecate multiple times per day, more often during times of grazing and ranging.^3^ Their faeces can be easily cleared from pastures and grazing areas by the owner of the premises; however, there is currently no standard of practice for collecting faecal waste upon each horse strolling/riding tour; hence, faecal droppings are normally abandoned in the environment and can also be found around public areas. According to recent reports, 56 zoonotic pathogens were identified in horses, of which over 40% were bacteria (eg, Methicillin-resistant *Staphylococcus* spp., *Salmonella* and *Escherichia coli – E. coli*) and 9% were protozoa (eg, *Cryptosporidium* and *Giardia*).^4^ *Campylobacter* spp. (eg, *Campylobacter jejuni – C. jejuni*) were predominantly detected in sick equines but rarely found in healthy horses.^5,6^ However, there is growing evidence of their presence in healthy equines across the world.^7,8^

According to the World Health Organization, *Campylobacter* is the most common bacterial pathogen causing human gastroenteritis.^9^ This has also been reflected in the Norwegian Surveillance System for Communicable Diseases.^10^ Moreover, it was identified as the most common bacterial cause of all reported waterborne outbreaks in Norway between 1998 and 2002.^11^ The largest waterborne campylobacteriosis outbreak occurred on a Norwegian island (Askøy) in summer 2019. It led to over 2000 illnesses and 76 hospitalizations and was caused by animal faecal pollution, to which horses were the major contributor.^12^ At that time, there were no official reports on local equine diseases; therefore, it is possible that healthy horses act as important vehicles of *Campylobacter* spp.

Based on the case of the largest waterborne campylobacteriosis outbreak in Norway, we hypothesize that healthy equines are carriers of enteropathogens, including *Campylobacter*. To gain a better and renewed insight into the ecological emergence of equine pathogens, we devised a pilot study aimed at stochastic screening examination of faecal droppings of healthy Norwegian horses to detect and quantify the enteropathogens most relevant to human health using genetic marker-based quantitative PCR (qPCR). The outcomes of this work contribute with new data to the zoonotic pathogen profile of healthy horses and can be further used in assessing potential health risks, environmental impacts and ecological threats.

## Materials and Methods

### Sampling campaign

Horse faecal samples were collected in May 2021 from 23 healthy individuals raised in 3 different properties scattered at various locations of Eastern Norway. The horses’ owners agreed to the sampling whilst keeping detailed information anonymously. Thus, the name of the involved stable, horse breed and age are reserved from disclosure in this study. However, general information is available, such as, all horses were adults and composed of 11 mares and 12 stallions. Sampling was carried out in the morning on fresh faecal droppings (none were loose, liquid or watery; all were in the form of solid faeces). The faecal material was collected in individual sampling plastic bags and cold transported to the lab on the same day. Upon arrival at the lab, 1.5 g was weighed out from each individual dropping sample and processed for faecal DNA extraction.

### Horse faecal DNA extraction

Triplicate faecal samples from each horse were subjected to DNA extraction using the DNeasy PowerLyzer kit (Qiagen GmbH, Hilden, Germany). DNA purification was carried out according to the manufacturer’s protocol with minor modifications to the bead beating step, where faecal samples with beads were homogenized on a Bead Mill MAX (VWR, Radnor, PA, USA) for 40 seconds at 6 m per sec for 2 cycles to ensure thorough homogenization. The yielded DNA was eluted in 100 µL elution buffer provided in the kit. DNA concentration and integrity were measured on a mySPEC Spectrophotometer (VWR, Radnor, PA, USA). The final concentration of purified DNA ranged from 40 to 100 ng·µL^−1^ with both 260/280 and 260/230 ratios between 1.8 and 2.0.

### Quantitative PCR analyses of enteropathogens in equines

The detection and quantification of selected bacterial enteropathogens *C. jejuni*, *Enterococcus faecalis* (*E. faecalis*), *Salmonella enterica* serovar Typhimurium (*S.* Typhimurium) and *Clostridium perfringens* (*C. perfringens*) were performed in duplicate on a Bio-Rad CFX Connect Real-Time PCR Detection System (Irvine, CA, USA) using species-specific DNA markers with 100% sensitivity and specificity. Detailed information on marker establishment and the sequences of the primers and probes were described in depth previously^13^ and are presented in Table 1. In addition, 2 more markers for protozoan pathogens *Cryptosporidium parvum* (*C. parvum*) and *Giardia lamblia* (*G. lamblia*), were developed for this study. Both new markers were sequence designed and verified in silico. The final marker genes were chemically synthesized (Thermo Fisher Scientific GENEART GmbH, Regensburg, Germany) and confirmed by sequencing. The plasmids carrying the target genes were molecular cloned into *E. coli* K12 OmniMAX™ 2 T1R (Thermo Fisher Scientific, Waltham, MA, USA). The sequence-validated clones were used for qPCR testing and optimization. In particular, the primers and probe for *C. parvum* were specifically designed, tested and established in this study. Among 3 designed sets of primers and probe combinations, the optimum one that exhibited the best qPCR performance with the highest sensitivity and specificity was identified and selected for sample examination. After adequate technical testing and optimization, assays with 95% to 100% PCR efficiency and linearity above 0.99 for the resulting standard curve were achieved and used for downstream pathogen detection. The detection limits for all markers tested were at 1.3 to 9.7 copies per reaction after optimization of each assay (ie, 1.3 copies for *C. jejuni*, 3.2 copies for *C. parvum*, 4.6 copies for *S.* Typhimurium, 8 copies for *E. faecalis*, 8 copies for *C. perfringens* and 9.7 copies for *G. lamblia*). Sequence information for the primers and probes used in qPCR assays for *C. parvum* and *G. lamblia* is shown in Table 1.

**Table 1.** Primers (F – forward primer, R – reverse primer) and probes (P – TaqMan probe) used for qPCR assays of the 6 target pathogens (*Campylobacter jejuni – C. jejuni*, *Salmonella enterica* serovar Typhimurium – *S.* Typhimurium, *Enterococcus faecalis – E. faecalis*, *Clostridium perfringens – C. perfringens*, *Giardia lamblia – G. lamblia* and *Cryptosporidium parvum – C. parvum*).

Pathogen	Sequences of Primes and Probes (5′–3′)	Genes	Reference
*C. jejuni*	F: CCATTAAAATTCTGACTTGCTAAAT	*hipO*	Gavrila et al.^13^
	R: ATGCTTGTGGTCATGATGGA		
	P: FAM-TTTTGCAGCAAGCAATAAAGAA-MGBNFQ		
*S.* Typhimurium	F: CCGCGATCTTTTTCTGATTC	*STM4497*	Gavrila et al.^13^
	R: GGAAAAGGACCACAAGTTCG		
	P: FAM-ACAGACGCGGTCAAATAACC-MGBNFQ		
*E. faecalis*	F: TGCTTGCACTCAATTGGAAA	*16S rRNA*	Gavrila et al.^13^
	R: CCCCTCTGATGGGTAGGTTA		
	P: FAM-GACGGGTGAGTAACACGTGG-MGBNFQ		
*C. perfringens*	F: TGAGACTTTTGCAGAGGAAAGA	*plc*	Gavrila et al.^13^
	R: GCCTCATTAGTTTTGCAACC		
	P: FAM-AAAGAACAGTATAAAATAAACACAGCA-MGBNFQ		
*G. lamblia*	F: GACGGCTCAGGACAACGGTT	*SSU rDNA*	Verweij et al.^14^
	R: TTGCCAGCGGTGTCCG		
	P: FAM-CCCGCGGCGGTCCCTGCTAG-MGBNFQ		
*C. parvum*	F: TTTTTCGTGTTAATAAATAGTGACTGC	*Cops2*	This study
	R: GTCGGAAGCCATAATGATCC		
	P: FAM-AGTAGGTGTGCTAGTCCAGGAA-MGBNFQ		

Pathogen qPCR examination was carried out in duplicate on a Bio-Rad CFX Connect Real-time PCR Detection System (Irvine, CA, USA). Twenty-microlitre qPCR reaction consisted of 10 µL of SsoAdvanced™ Universal Probes Supermix, 500 nM of each primer, 250 nM 5′-FAM probe and sterile nuclease-free H_2_O. The thermocycling conditions were 95°C for 3 minutes, followed by 40 cycles at 95°C for 15 seconds and 60°C for 30 seconds. The standard curve was constructed using 10-fold serial dilutions of plasmids carrying the marker gene (from 10^6^ to 10^0^ copies·µL^−1^). Target pathogen quantification was estimated using CFX Manager Version 3.1 (Bio-Rad, Irvine, CA, USA) on raw data derived from the qualified assay (ie, amplification efficiency ranging from 90% to 100% and regression rate above 0.99).

### Ethics statement

This pilot study was carried out in accordance with the general guidelines for research ethics in Norway.^15^ The research conducted was neither a medical or health research as defined by the Norwegian Health Research Act^16^ nor a clinical trial as defined by the International Committee of Medical Journal Editors.^17^ Animal faecal material was sampled on private properties. The sampling was agreed with the properties’ owners. The collection of samples was conducted in a manner that respected all common activities of the animals. Overall, all concerns over voluntariness, anonymity, confidentiality and impartiality were strictly respected throughout the entire study and ethical approvals for the research purpose were not requested according to the Norwegian National Research Ethics Committees.^15^

## Results and Discussion

From the screening examination of 6 targetted enteropathogens in the faeces of healthy equines (n = 23), the majority (14 horses) were found to carry 2 or 3 pathogens, while 4 carried only one pathogen. Moreover, 4 samples were positive for 4 pathogens, and only one was positive for all except *C. parvum.* The detailed outcomes of this pilot study are shown in Table 2.

**Table 2.** Copy numbers of the 6 target pathogens (*Campylobacter jejuni* – *C. jejuni*, *Enterococcus faecalis* – *E. faecalis*, *Salmonella enterica* serovar Typhimurium – *S*. Typhimurium, *Clostridium perfringens* – *C. perfringens*, *Cryptosporidium parvum* – *C. parvum* and *Giardia lamblia* – *G. lamblia*) in 1 g individual horse faeces (n = 23). Pathogen rate – number of detected pathogens in each individual sample. Positivity rate – number of positive cases for each respective pathogen among all examined horses (H – horses, ND – not detected).

Horses	*C. jejuni*	*E. faecalis*	*S.* Typhimurium	*C. perfringens*	*C. parvum*	*G. lamblia*	Pathogen rate
H1	ND	ND	ND	*1.19E* + 05	ND	ND	1/6
H2	ND	*1.01E* + 04	ND	*6.69E* + 03	ND	*7.73E* + 06	3/6
H3	ND	*7.71E* + 03	ND	*5.94E* + 03	ND	ND	2/6
H4	*1.33E* + 00	*2.87E* + 03	ND	*3.69E* + 05	ND	*6.14E* + 06	4/6
H5	*3.33E* + 01	*8.81E* + 03	ND	*2.74E* + 04	*6.21E* + 02	ND	4/6
H6	ND	ND	ND	*3.81E* + 04	ND	*3.34E* + 07	2/6
H7	ND	*5.77E + 03*	ND	ND	ND	ND	1/6
H8	ND	ND	ND	*3.84E* + 03	*6.47E* + 02	ND	2/6
H9	*1.83E* + 01	*9.85E* + 03	ND	ND	*1.05E* + 02	ND	3/6
H10	ND	*6.89E* + 03	ND	*2.64E* + 03	ND	ND	2/6
H11	ND	ND	*1.56E* + 02	*3.84E* + 03	ND	ND	2/6
H12	ND	*8.79E* + 03	ND	*1.76E* + 04	ND	ND	2/6
H13	ND	ND	ND	*4.76E* + 03	ND	ND	1/6
H14	ND	*3.79E* + 03	*1.65E* + 01	ND	*2.65E* + 02	ND	3/6
H15	*2.20E* + 01	ND	ND	*5.35E* + 04	*2.27E* + 02	*4.62E* + 06	4/6
H16	ND	*8.08E* + 03	ND	*9.59E* + 02	*1.90E* + 02	ND	3/6
H17	*3.30E* + 01	*7.41E* + 03	ND	*6.13E* + 03	*9.93E* + 02	ND	4/6
H18	*2.64E* + 01	*4.62E* + 03	*3.21E* + 01	*9.07E* + 02	ND	*1.65E* + 05	5/6
H19	ND	*8.53E* + 03	*3.94E* + 01	*2.14E* + 03	ND	ND	3/6
H20	ND	ND	ND	ND	*5.26E* + 02	ND	1/6
H21	ND	ND	ND	*1.48E* + 05	ND	*2.34E* + 06	2/6
H22	ND	ND	*2.82E* + 01	*1.59E* + 03	ND	ND	2/6
H23	ND	*8.32E* + 03	ND	*4.51E* + 03	ND	*1.35E* + 03	3/6
Average concentration	*2.24E* + 01	*7.25E* + 03	*5.45E* + 01	*4.30E* + 04	*4.47E* + 02	*7.77E* + 06	
Positivity rate	6/23	14/23	5/23	19/23	8/23	7/23	

The protozoan parasites *C. parvum* and *G. lamblia* were detected in 8 and 7 horses, respectively. However, a very high abundance of *G. lamblia* was found in horse faeces, with 7.77 × 10^6^ copies, in contrast to only 4.47 × 10^2^ copies of *C. parvum. G. lamblia* is a common widespread intestinal parasite of a broad range of hosts, such as humans as well as domestic and wild animals. It was first reported as a parasite of horses in South Africa in 1921^18^ and was later also found in Chinese grazing horses^19^ and in 17.4% of examined horses from Colombia.^20^ However, the infected equines rarely show any clinical signs. The case is similar with *C. parvum*, which was reported to have a prevalence of 25.3% to 37.8% in equines worldwide.^21^ Many horses can become asymptomatic carriers of these protozoan parasites without manifesting significant clinical symptoms (eg, abdominal pain, dehydration and weight loss).^22^ However, they may transmit these pathogens to humans, for instance, about 90% of human cryptosporidiosis cases were caused by *C. parvum* and *Cryptosporidium hominis* (*C. hominis*).^21^

Among the tested bacterial enteropathogens, *C. perfringens* was detected in the faeces of 19 horses, representing the highest prevalence (19/23), with an average concentration of 4.3 × 10^4^ copies·g^−1^ of faecal material (Table 2). It is worth noting that only strains of *C. perfringens* that produce some toxins can cause disease in humans. This bacterium has been found normally (commensal) in the gastrointestinal (GI) tract of equines and has been identified in both healthy and sick individuals.^23,24^ This phenomenon was studied earlier by Costa et al.^25^ using high-throughput sequencing to compare the faecal microbiota of healthy horses and those with colitis, and the results indicated a high abundance of the *Clostridium* genus among healthy horses. They revealed that *Clostridia* are a key component of the equine intestinal microbiota and thus can predominate in healthy horses. Similar findings were recently reported by Paßlack et al.,^26^ indicating a dietary impact on the equine faecal microbial composition and a high relative abundance of *Clostridiales* in the faeces of healthy horses.

*E. faecalis* was detected in 14 horses at 7.25 × 10^3^ copies·g^−1^ (mean concentration), making it the second most prevalent enteropathogen in this study (Table 2). *Enterococci* are commensal bacteria residing in the GI tracts of almost all land animals; however, they can cause severe infections in humans^27^ and have become important nosocomial pathogens globally. In a recent study by Sukmawinata et al.^28^ on *Enterococci* isolate profiling, 16.4% of *E. faecalis* were detected in a yearly collection of 72 faecal samples from healthy Japanese foals. Moreover, they found that the detected *E. faecalis* carried antimicrobial resistance to various antibiotics, for example, oxytetracycline, kanamycin, gentamycin and chloramphenicol. Thus, they become clinically important pathogens responsible for life-threatening hospital-acquired infections.^29^ In this pilot study, we focussed primarily on zoonotic pathogen profiling in healthy equines, the associated antibiotic resistome in horses will thus be addressed in a following study, since it also has direct health implications.

*S.* Typhimurium was found only in the faeces of 5 horses at a very low average content (54.5 copies·g^−1^ faeces, Table 2). *Salmonella* spp. can cause a variety of clinical equine diseases, from self-limiting diarrhoea to acute colitis with haemorrhagic diarrhoea and endotoxemia^30^; however, many horses can act as subclinical carriers, that is, can carry *Salmonella* in their guts without shedding or being ill (asymptomatic shedders). The development of serious salmonellosis in horses depends on many factors, for example, the animal’s general health condition, the virulence of the pathogen and the amount of harboured *Salmonella*.^31^ In our study, all examined horses were in good form, and there was no report of any enteric diseases (eg, diarrhoea), thus we consider these horses as asymptomatic carriers of *Salmonella* and the other detected pathogens. This implies that they might not fall sick themselves but may transmit pathogens to others (including other animals and humans) and to the environment.

*C. jejuni* is a commensal bacterial member of the gut microbiota of many domesticated and wild animals. However, *Campylobacter* is 1 of 4 global causative agents of diarrhoeal diseases.^9^ Among all species, *C. jejuni* and *Campylobacter coli* (*C. coli*) are considered as the leading causative agents of human infectious disease campylobacteriosis. In the current study, we identified 6 positive cases carrying a low content of *C. jejuni* (on average 22.4 copies·g^−1^ faeces, Table 2). Worldwide, there are few reports indicating that healthy and sick equines carry *Campylobacter* spp.^5,8,32-34^ and the latest published data from Norway^35^ described few cases but rather, sporadic isolation of *Campylobacter* from horses (with unknown health status). Of note, different examination methods can lead to varied analysis results. Advanced molecular approaches are expected to deliver better detection resolution and higher sensitivity compared with traditional (old) culture-based methods, largely due to the fact that most pathogens are at low abundance and hard to culture. Even though *C. jejuni* is rarely detected and is present at low abundance, the risks to human and environment health cannot be overlooked. Thus, potential disease transmission concerns have arisen directly by close contact or indirectly by faecal pollution of the environment.^8^ However, full awareness of horse-related health risks is rather poor, and many public health professionals are underinformed.^4^ In a Canadian survey conducted for 214 public health inspectors, over two-thirds of professionals considered diseases transmitted from horses having no or some impact on public health.^36^ Another similar survey undertaken in New Zealand revealed that only 31% equine properties had any type of biosecurity protocols for the visitors.^37^ Apparently, there are significant knowledge gaps related to equine zoonoses and their transmission routes (eg, faecal shedding of pathogens), environmental pollution risks (eg, faecal contamination of grazing areas and water bodies) and ecological consequences of faecal contamination (eg, deterioration of ecosystem biodiversity and functionality). Thus, proactive dissemination of updated knowledge and information to a broader public is of vital importance to improve overall public awareness and establish well-informed biosecurity measures.

## Conclusions and Remarks

This pilot study provides evidence that healthy equines can carry *C. jejuni* and other zoonotic pathogens, which may pose health risks to humans and animals, and jeopardise environmental protection. None of the tested faecal samples was pathogen free. Given that horse-related recreational activities are very popular worldwide, it is imperative to establish information-guided decisions and implement intensified socio-ecological biosecurity procedures among all horse practitioners. Jointly, there is an urgent need for the environmental sectors to reinforce management efforts on surveillance, control and mitigation of zoonoses transmitted through environmental matrices.

To extend our knowledge scope on the potential human and ecological threats from zoogenic faecal contamination, further investigations on a larger scale (eg, including a higher number of equines) will follow. Given that antimicrobial resistance is increasingly associated with zoonotic enteropathogens, the antibiotic resistome of faecally shedded pathogens will also be addressed and explored.
